# *Streptococcus intermedius* Bacteremia and Liver Abscess following a Routine Dental Cleaning

**DOI:** 10.1155/2014/954046

**Published:** 2014-08-13

**Authors:** Lachara V. Livingston, Elimarys Perez-Colon

**Affiliations:** Department of Internal Medicine, Morsani College of Medicine, University of South Florida, 17 Davis Boulevard, Suite 308, Tampa, FL 33606, USA

## Abstract

*Streptococcus intermedius* is a member of the *Streptococcus anginosus* group of bacteria. This group is part of the normal flora of the oropharynx, genitourinary, and gastrointestinal tracts; however, they have been known to cause a variety of purulent infections including meningitis, endocarditis, and abscesses, even in immunocompetent hosts. In particular, *S. intermedius* has been associated with the development of liver and brain abscesses. There have been several case reports of *S. intermedius* liver abscesses with active periodontal infection. To our knowledge, however, there has not been a case following a routine dental procedure. In fact, the development of liver abscesses secondary to dental procedures is very rare in general, and there are only a few case reports in the literature describing this in relation to any pathogen. We present a rare case of *S. intermedius* bacteremia and liver abscess following a dental cleaning. This case serves to further emphasize that even routine dental procedures can place a patient at risk of the development of bacteremia and liver abscesses. For this reason, the clinician must be sure to perform a detailed history and careful examination. Timely diagnosis of pyogenic liver abscesses is vital, as they are typically fatal if left untreated.

## 1. Introduction

*Streptococcus intermedius* is a member of the* Streptococcus anginosus* group, also known as the* Streptococcus milleri* group. Members of this group include* S. intermedius, S. anginosus, *and* S. constellatus* [1]. They were originally grouped into one species but were later able to be separated based on their variable expression of Lancefield group antigens and hemolytic activity. However,* S. intermedius *is nonhemolytic and phenotypically ungroupable [2].

Although they are infrequent pathogens and are found as part of the normal flora of the oropharynx, genitourinary tract, and gastrointestinal tract [3], members of the* Streptococcus anginosus* group have been implicated in a variety of purulent infections and abscess formation. This includes those of the brain, meninges, heart, sinuses, liver, lungs, spleen, peritoneum, pelvis, and appendix [1, 3–5]. It has been suggested in some reports that infections by these bacteria are increased in patients with multiple comorbidities, malignancy, and diabetes [1].

The first cases of* S. anginosus* group causing hepatic abscesses were reported in 1975 [6]. A subsequent study in 1981 found this group of bacteria to be the most common cause of hepatic abscesses [7], and* S. intermedius* was the species most often isolated from hepatic abscesses in a prospective 1998 study [8]. Members of the* S. anginosus* group have frequently been isolated from dental abscesses, implicating this as one of the possible sources of many of the group's metastatic purulent infections. In a 1990 study by Whiley et al.,* S. intermedius* was the group member most commonly found in dental plaques and in association with hepatic and brain abscesses. However,* S. intermedius* was not frequently associated with actual infections of the oral cavity, making a causal relationship difficult to establish [2].

A literature search was performed in order to identify cases of liver abscess due to* S. intermedius* in the presence of active oral infection or prior dental procedures. There are several cases of reported* S. intermedius* bacteremia as the causal agent of liver abscesses in the presence of active oral infection; however, none were found in the case of prior dental procedures. A 2006 case report by Wagner et al. reported on the case of brain and liver abscesses due to coexisting severe periodontal disease and bacteremia with* S. intermedius* being cultured from all four sources [9]. More recently, a 2009 case report by Ng and Mukhopadhyay documented a case of* S. intermedius* bacteremia causing liver abscess and septic shock immediately following a partial tooth extraction for a periodontal abscess [3]. Similarly, Neumayr et al. reported a case of multiple liver abscesses caused by* S. intermedius* in a patient with a concurrent pyogenic dental infection in a 2010 case report [4].

Our case illustrates a rare case of* S. intermedius* bacteremia and liver abscess following a routine dental cleaning.

## 2. Case Presentation

A 65-year-old African-American man with a history of hypertension, reflux disease, allergic rhinitis, and peptic ulcer disease presented to the emergency department with dull, nonradiating, right-sided abdominal pain, watery diarrhea, nausea, and poor appetite for seven days. He also complained of a thirteen-pound weight loss over the eleven months prior to admission. He denied chest pain, shortness of breath, rashes, dysuria, melena, hematochezia, arthralgias, or myalgias. The patient denied tobacco, alcohol, or illicit drug use, and he had no recent domestic or exotic travel. He admitted to having a blood transfusion in 1975. He was also evaluated by an oral surgeon and had a tooth extraction approximately eleven months prior to hospitalization, in addition to a dental cleaning 1.5 months ago. No prophylactic antibiotics were given prior to these procedures.

Family history was significant for a mother with coronary disease and a sister with diabetes. He reported having four healthy children, living alone, and working from home. Medications included fluticasone nasal spray daily, losartan 100 mg daily, ranitidine 150 mg twice daily, and pantoprazole 40 mg twice daily. He develops a diffuse rash with penicillin use.

Of note, he was treated for cholecystitis and choledocholithiasis after presenting with obstructive jaundice, transaminitis, and abdominal pain over 1.5 years earlier. MRCP showed multiple stones within the extrahepatic biliary ductal system consistent with choledocholithiasis and mild dilation of both the intra- and extrahepatic bile ducts suggestive of biliary obstruction. He was afebrile and there was no evidence of acute cholecystitis on imaging, but he was treated prophylactically with ciprofloxacin and metronidazole due to positive Murphy's sign and transaminitis (AST 260 IU/L, ALT 396 IU/L, total bilirubin 7.0 mg/dL). Gastroenterology performed two successive ERCPs due to difficulty with cannulation related to obstructive stones. The first entailed partial sphincterotomy and prophylactic 5Fr 3 cm pancreatic duct (PD) stent placement. The following day, the PD stent was removed, the common bile duct (CBD) was dilated to 12 mm, and the sphincterotomy was extended to allow the extraction of four stones. Occlusion cholangiogram revealed one retained stone in the cystic duct, and a 10F 5 cm stent was placed in the CBD to ensure proper drainage. A trickle of blood was observed at the upper margin of the sphincterotomy but abated with the spraying of epinephrine. Laparoscopic cholecystectomy was performed two days later, and he was discharged home on postoperative day number one.* H. pylori* stool antigen was also found to be positive, so he was discharged to complete a two-week course of triple antibiotic therapy (clarithromycin 500 mg po twice daily, omeprazole 20 mg po twice daily, and metronidazole 500 mg po twice daily). Repeat ERCP was performed approximately a month later with the removal of the single residual stone and extraction of the CBD stent without complication.

On his presentation to our team, temperature was 102.8 F, pulse 85, respirations 16, and blood pressure 116/66 mmHg. Physical exam revealed a well-nourished man in no acute distress that appeared in his stated age. Eyes were anicteric. There were multiple silver and gold tooth fillings but no evidence of dental caries, periodontitis, or oral abscess was found. Cardiovascular exam was negative for murmurs, and there was no lower extremity edema. Lungs were clear to auscultation bilaterally. Abdomen was obese without fluid wave. There was moderate tenderness to palpation at the right upper quadrant with negative Murphy's sign. There was also no rebound, guarding, hepatosplenomegaly, rash, or jaundice.

**Figure 1:**
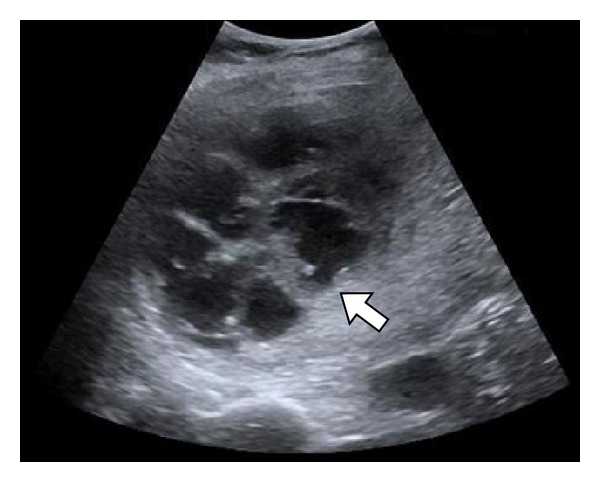
Right upper quadrant ultrasound showing a complex hepatic lesion with multiple internal septations (arrow).

Laboratory data showed WBC 23.2 k/*μ*L, hemoglobin 12.2 gm/dL, platelets 442 k/*μ*L, creatinine 1.6 mg/dL, AST 138 IU/L, ALT 136 IU/L, alkaline phosphatase 176 *μ*/L, total bilirubin 2.4 mg/dL, albumin 2.5 gm/dL, lipase 90 *μ*/L, amylase 58 *μ*/L, and negative urinalysis, and coagulation studies were within normal limits. Acute hepatitis panel was negative, and urine drug screen was negative other than for opiates which he received in the emergency department. Right upper quadrant ultrasound (Figure 1) showed a homogenous liver and a complex, predominant cystic lesion measuring 10.2 × 7.4 × 9.4 cm within the right hepatic lobe demonstrating multiple internal thickened septations with color Doppler flow as well as debris. The common bile duct was within normal limits, and the gallbladder was surgically absent. Blood cultures were drawn, and he was empirically placed on ciprofloxacin 400 mg intravenous (IV) every 12 hours and metronidazole 500 mg IV every 8 hours due to history of penicillin allergy without anaphylaxis.

**Figure 2:**
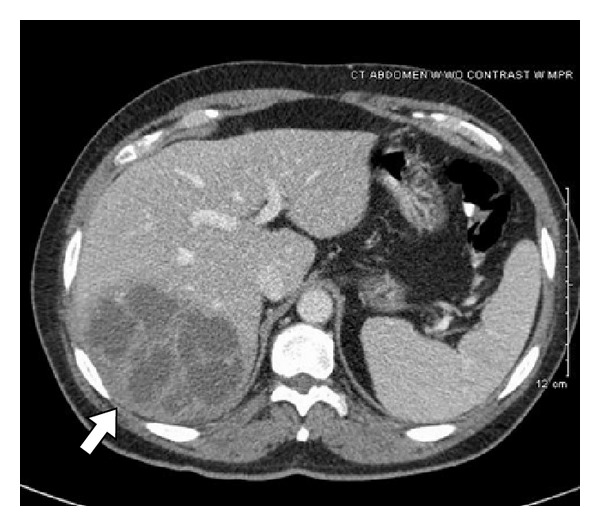
CT venous phase showing large hepatic abscess (arrow).

Contrasted CT imaging was initially deferred so that intravenous fluids could be given to improve the patient's creatinine. After the creatinine normalized, three-phase CT of the abdomen with delayed imaging (Figure 2) was obtained demonstrating a 13.0 × 10.2 × 7.8 cm cystic, heterogeneously arterially enhancing lesion involving segments VI and VII of the liver with multiple enhancing septations with peripheral enhancement on the arterial phase and partial venous and delayed phase washout. There was also a focus of gas within the porta hepatis. Findings were thought to be most consistent with hepatic abscess; however, necrotic neoplasm could not be excluded.

A CT-guided drain was placed with the expression of blood-tinged, purulent fluid which was sent for multiple studies. In the meantime, blood cultures which were obtained on admission resulted positive with gram positive cocci in pairs and chains, so antibiotics were switched to ceftriaxone 2 gm IV daily with metronidazole 500 mg IV every 8 hours. In addition, AFP < 2.0 ng/mL and* Entamoeba histolytica* IgG was negative. Both blood and fluid cultures eventually grew* Streptococcus intermedius. *This was isolated using blood and chocolate agar medium and a VITEK 2 gram positive card. Fungal culture was negative. Susceptibility studies revealed sensitivities and MIC values as follows: cefotaxime 0.019, ceftriaxone 0.125, penicillin 0.064, and vancomycin 1.0. Transthoracic echocardiogram was later performed showing ejection fraction 60–65% and no discrete valvular lesions. He refused to submit to HIV testing.

The patient's clinical status improved, and repeat blood cultures were negative. He was discharged with the hepatic drain in place with plans for removal in outpatient clinic in one week's time. A PICC line was inserted, and he was placed on ceftriaxone 2 gm IV daily to complete a four-week course of intravenous therapy.

## 3. Discussion

In conclusion, liver abscesses are most frequently associated with disorders of the biliary tract and can include biliary stone disease, malignancy, and congenital disorders. Direct extension from intra-abdominal infections and hematogenous spread are also possibilities [10]. The most common offending organisms are gram-negative aerobes, with* Escherichia coli* being the most common [11].

Dental procedures are a very rare etiology of pyogenic liver abscesses in general, with only a few cases being reported in the literature. In 1987, Tweedy and White reported a case of multiple liver abscesses due to* Fusobacterium nucleatum* following “extensive dental work” in a man with suspected primary immunodeficiency [12]. Schiff et al. (2003) also reported a case of a previously healthy woman presenting with multiple liver abscesses following a root canal filling one week prior [11], and more recently, Gungor et al. (2012) reported a case of “Streptococcal subspecies” causing liver abscess ten days following a dental prosthesis implantation in a patient with diabetes [13]. To our knowledge, this is the first reported case of pyogenic liver abscess related to* S. intermedius* following a routine dental cleaning.

The presence of* S. anginosus* group bacteremia should not only alert the clinician to the possibility of underlying abscess as a source for infection.* S. intermedius* bacteremia and liver abscess should raise suspicion for recent history of dental manipulation in addition to active oral infection. In our case, we believe that this patient's prior dental cleaning caused bacteremia and seeding of the liver via a hematogenous route. Lack of other identifiable sources of infection and the isolation of* S. intermedius* from both blood and liver abscess samples support this conclusion. Given that his ERCP and cholecystectomy were more than one year prior to his presentation, it is unlikely that mucosal injury related to these procedures was linked to this presentation. In addition, his dental extraction occurred almost a year prior to presentation and is thus unlikely to have contributed. Even routine dental cleanings can predispose patients to the development of bacteremia and hepatic abscess, so detailed history taking is essential in addition to thorough examination of the oropharynx. Prompt diagnosis and treatment are imperative, as pyogenic liver abscesses are almost uniformly fatal otherwise.
